# Unravelling the diversity of *Posthodiplostomum* Dubois, 1936 (Trematoda: Diplostomidae) in fish-eating birds from the Neotropical region of Mexico, with the description of a new species

**DOI:** 10.1017/S0031182024000970

**Published:** 2024-09

**Authors:** Marcelo Tonatiuh González-García, Alejandra López-Jiménez, Mirza Patricia Ortega-Olivares, Ana Lucia Sereno-Uribe, Gerardo Pérez-Ponce de León, Martín García-Varela

**Affiliations:** 1 Departamento de Zoología, Instituto de Biología, Universidad Nacional Autónoma de México, Avenida Universidad 3000, Ciudad Universitaria, Ciudad de México, CP, México; 2 Posgrado en Ciencias Biológicas, Universidad Nacional Autónoma de México, Avenida Universidad 3000, Ciudad Universitaria, Ciudad de México, CP, México; 3 Departamento de Biología Evolutiva, Facultad de Ciencias, Universidad Nacional Autónoma de México, Avenida Universidad 3000, Ciudad Universitaria, Ciudad de México, CP, México; 4 Departamento de Sistemas y Procesos Naturales, Escuela Nacional de Estudios Superiores Unidad Mérida, Yucatán, CP, México

**Keywords:** ardeidae, laridae, molecular markers, morphology, phylogeny, *Posthodiplostomum*

## Abstract

Adults of the genus *Posthodiplostomum*, Dubois, 1936 are parasites of fish-eating birds, mainly of the family Ardeidae, and are globally distributed. The genus currently comprises 35 species, although recent molecular evidence has shown that the diversity of the genus is underestimated since several candidate species have been recognized. In the Neotropical region of Mexico, at least 6 *Posthodiplostomum* lineages have been detected with metacercaria stages recovered from unrelated fish hosts. Here, we obtained adult specimens of *Posthodiplostomum* from 6 fish-eating birds representing 2 families (*Butorides virescens*, *Ardea herodias*, *Nycticorax nycticorax*, *Tigrisoma mexicanum* – Ardeidae, and *Rynchops niger* and *Leucophaeus atricilla* – Lariidae) from 4 localities in southern Mexico. Specimens were sequenced for 2 nuclear (28S and ITS1–5.8S–ITS2) and 1 mitochondrial (*cox1*) molecular marker. Phylogenetic analyses allowed us to link metacercariae and adult specimens and recognized a lineage, which was described morphologically. The new species can be distinguished from its congeners by its prosoma morphology and body size; this is the first described species in the Neotropical region of Mexico. Additionally, new host and locality records for *P. macrocotyle* and *P. pricei* are presented, expanding their geographical distribution range in the Americas.

## Graphical abstract

**Figure:**
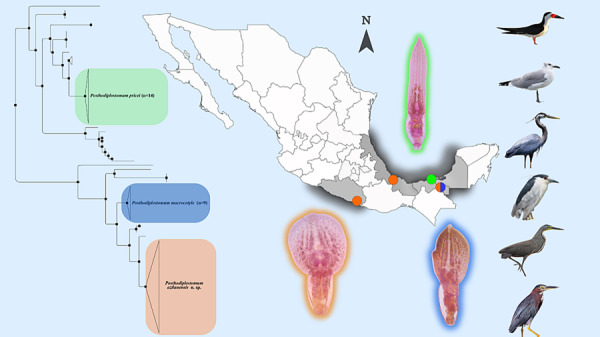

## Introduction

Diplostomidae Poirier, 1886, is a large and globally distributed family of digeneans whose adults are found in the intestines of birds and mammals (Niewiadomska, 2002; Heneberg *et al*., 2020). Among diplostomids, the genus *Posthodiplostomum* Dubois, 1936, has been investigated in numerous studies related to their taxonomy, ecology, host–parasite relationships and pathogenicity (e.g. Dubois, 1970; Niewiadomska, 2002; López-Hernández *et al*., 2018; Achatz *et al*., 2021). A recently published study on the diversity of the subfamily former Crassiphialinae Sudarikov, 1960, through molecular data proposed the synonymy of the genera *Ornithodiplostomum* Dubois, 1936 and *Mesoophorodiplostomum* Dubois, 1936 with *Posthodiplostomum* (Achatz *et al*., 2021). According to this new taxonomic reorganization, the genus *Posthodiplostomum* currently contains 35 species; most species in the genus, as adults, are parasites of fish-eating birds of the family Ardeidae Leach (Dubois, 1970; Niewiadomska, 2002; López-Hernández *et al*., 2018; Achatz *et al*., 2021). The database of DNA sequences from *Posthodiplostomum* has increased steadily in recent years with the availability of sequence data from global sources, expanding our knowledge of species diversity, classification and biogeography. Nevertheless, assembling a comprehensive molecular database has been challenging because various authors have sequenced different nuclear regions, e.g. the D2–D3 or D1–D3 domains of the large (28S) or small subunit (18S), the transcribed spacers (ITS1–5.8S–ITS2), and different regions of the mitochondrial gene as the first region the 5′ beginning (typical barcoding region) or second region the 3′end of cytochrome oxidase (*cox1*) (see Locke *et al*., 2010; Nguyễn *et al*., 2012; Kvach *et al*., 2017; Stoyanov *et al*., 2017; Boone *et al*., 2018; López-Hernández *et al*., 2018; Hoogendoorn *et al*., 2019; Sokolov and Gordeev, 2020; Achatz *et al*., 2021; Duan *et al*., 2021; Pernett *et al*., 2022; Pérez-Ponce de León *et al*., 2022).

The only species of *Posthodiplostomum* known to parasitize fish and fish-eating birds across Mexico was *P. minimum* (McCallum, 1921) Dubois, 1936 (Pérez-Ponce de León *et al*., 2007). However, extensive sampling of metacercariae and adults of *Posthodiplostomum* and the use of molecular tools allowed us to uncover a large species diversity in the genus. For example, Pérez-Ponce de León *et al*., 2022 identified 6 genetic lineages in what was once considered a single species. The metacercariae of *P. minimum* have been reported from 109 fish species and adults from 7 species of fish-eating birds (Pérez-Ponce de León *et al*., 2007, 2022). However, no molecular data are available for adults, preventing the establishment of a link between larval forms and adults and the recognition of candidate species instead of only genetic lineages (Locke *et al*., 2010; López-Hernández *et al*., 2018; Achatz *et al*., 2021; Pérez-Ponce de León *et al*., 2022).

Here, we filled out the knowledge gap concerning the molecular diversity and host associations of *Posthodiplostomum* in fish-eating birds across the Neotropical region of Mexico, employing an integrative taxonomic approach, we generated sequences of the large subunit (28S), internal transcribed spacers (ITS1–5.8S–ITS2) from nuclear DNA, and cytochrome c oxidase subunit 1 (*cox1*) from mitochondrial DNA from adult specimens of *Posthodiplostomum*. The main objectives of this study were to explore the molecular diversity of *Posthodiplostomum* in this region, to establish molecular links between newly sequenced adults and previously identified genetic lineages of metacercariae, and to expand our understanding of host and locality records for the genus.

## Materials and methods

### Specimen collection and morphological analyses

**Figure 1.**
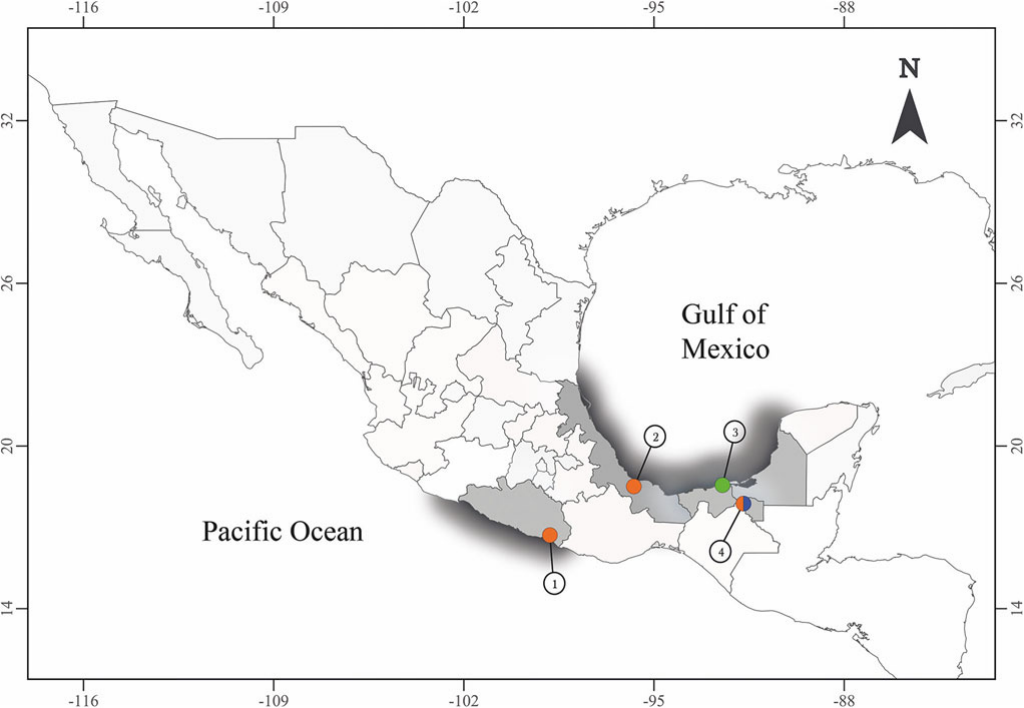
Sampling collection in Mexico. (1) Marquelia, Guerrero (16°35′41.5″N, 98°50′38″W), (2) Tlacotalpan, Veracruz (18°36′0″N, 95°39′0″W), (3) Nuevo Campechito, Champeche (18°38′55.849″N, 92°28′2.578″W), (4) Emiliano Zapata, Tabasco (17°46′29.1″N, 91°44′24.9″W). The colours represent the species recovered; in orange, *Posthodiplostomum aztlanensis* n. sp., in green *Posthodiplostomum pricei* and in blue *Posthodiplostomum macrocotyle.*

Seven specimens of fish-eating birds representing 2 families, Ardeidae and Laridae Rafinesque were collected in 4 localities in Mexico (Fig. 1; Table 1). Birds were identified following Howell and Webb (1995), and the American Ornithologist’ Union (1998). Adult diplostomids morphologically identified as *Posthodiplostomum* spp., were obtained from the intestines of 6 avian hosts. Diplostomids were heat-killed with distilled water, and preserved in 100% ethanol for DNA analyses. Additionally, specimens were fixed in hot 4% formalin for scanning electron microscopy studies.

**Table 1.** Summary data for the taxa used in the phylogenetic analyses

				GenBank accession number				
Taxa	Host species	Locality		LSU	ITS	1^st^ *cox1*	2^nd^ *cox1*	Source
*Posthodiplostomum pricei*	*Rynchops niger*	México	A	**PP718620-PP718631**	**PP718657-PP718665**	**PP724758-PP724759**	**PP724758-PP724759**	**This study**
*Posthodiplostomum pricei*	*Larus argentatus*	Canada	A			HM064859		Locke *et al*. (2010)
*Posthodiplostomum pricei*	*Morone americana*	Canada	M		HM064959, HM064960	HM064860, HM064861		Locke *et al*. (2010)
*Posthodiplostomum pricei*	*Larus delawarensis*	Canada	A			HM064862, HM064864		Locke *et al*. (2010)
*Posthodiplostomum pricei*	*Larus delawarensis*	USA	A	MZ710972, MZ710973		MZ707199, MZ707200		Achatz *et al*. (2021)
*Posthodiplostomum macrocotyle*	*Ardea herodias*	México	A	**PP718632-PP718635**	**PP718666**			**This study**
*Posthodiplostomum macrocotyle*	*Tigrisoma mexicanum*	México	A	**PP718636, PP718637**	**PP718668, PP718669**			**This study**
*Posthodiplostomum macrocotyle*	*Leucophaeus atricilla*	México	A	**PP718638**	**PP718667**			**This study**
*Posthodiplostomum macrocotyle*	*Busarellus nigricollis*	Brazil	A	MZ710958, MZ710959		MZ707188, MZ707189		Achatz *et al*. (2021)
*Posthodiplostomum macrocotyle*	*Parachromis managuensis*	Puerto Rico	M			OP071174, OP071176		Pernett *et al*. (2022)
*Posthodiplostomum aztlanensis* n. sp.	*Butorides virescens*	México	A	**PP718600-PP718612**	**PP718639-PP718651**	**PP724755- PP724757**	**PP724755, PP724756**	**This study**
*Posthodiplostomum aztlanensis* n. sp.	*Nycticorax nycticorax*	México	A	**PP718613-PP718616**	**PP718652-PP718655**			**This study**
*Posthodiplostomum aztlanensis* n. sp.	*Tigrisoma mexicanum*	México	A	**PP718617-PP718619**	**PP718656**			**This study**
*Posthodiplostomum aztlanensis* n. sp.	*Ardea herodias*	México	A	**PP718598, PP718599**				**This study**
*Posthodiplostomum aztlanensis* n. sp.	*Poecilia* sp.	Honduras	M	**PP718597**	OK315782			**This study/**Pérez-Ponce de León *et al*. (2022)
*Posthodiplostomum aztlanensis* n. sp. ^a^	*Goodea atripinnis*	México	M		OK315754		OK314911, OK314912	Pérez-Ponce de León *et al*. (2022)
*Posthodiplostomum aztlanensis* n. sp. ^a^	*Gobiomorus maculatus*	Costa Rica	M		OK315788			Pérez-Ponce de León *et al*. (2022)
*Posthodiplostomum* sp. Lineage I	*Poecilia formosa*	México	M		OK315682		OK314873	Pérez-Ponce de León *et al*. (2022)
*Posthodiplostomum* sp. Lineage I	*Poecilia* sp.	México	M				OK314879	Pérez-Ponce de León *et al*. (2022)
*Posthodiplostomum* sp. Lineage I	*Poecilia catemaconis*	México	M		OK315685			Pérez-Ponce de León *et al*. (2022)
*Posthodiplostomum* sp. Lineage I	*Gambusia affinis*	México	M		OK315672			Pérez-Ponce de León *et al*. (2022)
*Posthodiplostomum* sp. Lineage II	*Goodea atripinnis*	México	M		OK315756			Pérez-Ponce de León *et al*. (2022)
*Posthodiplostomum* sp. Lineage II	*Skiffia lermae*	México	M		OK315769			Pérez-Ponce de León *et al*. (2022)
*Posthodiplostomum* sp. Lineage II	*Gambusia* sp.	México	M		OK315772			Pérez-Ponce de León *et al*. (2022)
*Posthodiplostomum* sp. Lineage II	*Pimephales promelas*	México	M		OK315774			Pérez-Ponce de León *et al*. (2022)
*Posthodiplostomum* sp. Lineage II	*Allotoca dugesii*	México	M				OK314916	Pérez-Ponce de León *et al*. (2022)
*Posthodiplostomum* sp. Lineage II	*Goodea atripinnis*	México	M				OK314909	Pérez-Ponce de León *et al*. (2022)
*Posthodiplostomum* sp. Lineage III	*Vieja* sp.	México	M		OK315706			Pérez-Ponce de León *et al*. (2022)
*Posthodiplostomum* sp. Lineage III	*Herichthys labridens*	México	M		OK315744		OK314904	Pérez-Ponce de León *et al*. (2022)
*Posthodiplostomum* sp. Lineage III	*Amatitlania siquia*	Nicaragua	M		OK315785			Pérez-Ponce de León *et al*. (2022)
*Posthodiplostomum* sp. Lineage IV	*Poecilia sphenops*	México	M		OK315724			Pérez-Ponce de León *et al*. (2022)
*Posthodiplostomum* sp. Lineage IV	*Profundulus punctatus*	México	M		OK315738, OK315741		OK314901, OK314902	Pérez-Ponce de León *et al*. (2022)
*Posthodiplostomum* sp. Lineage VI	*Vieja sp.*	México	M		OK315703			Pérez-Ponce de León *et al*. (2022)
*Posthodiplostomum cuticola*	*Alburnus alburnus*	Denmark	M		MW135136			Unpublished
*Posthodiplostomum cuticola*	*Squalius cephalus*	Turkey	M			MN701652		Unpublished
*Posthodiplostomum cuticola*	*Nycticorax nycticorax*	Ukraine	A	MZ710955		MZ707185		Achatz *et al*. (2021)
*Posthodiplostomum erickgreenei*	*Pandion haliaetus*	USA	A	MZ710956		MZ707186	MZ707186	Achatz *et al*. (2021)
*Posthodiplostomum eurypygae*	*Eurypyga helias*	Brazil	A	MZ710957		MZ707187		Achatz *et al*. (2021)
*Posthodiplostomum orchilongum*	*Ardea alba*	USA	A	MZ710964				Achatz *et al*. (2021)
*Posthodiplostomum orchilongum*	*Egretta caerulea*	USA	A	MZ710965, MZ710966		MZ707193		Achatz *et al*. (2021)
*Posthodiplostomum microsicya*	*Tigrisoma lineatum*	Brazil	A	MZ710960				Achatz *et al*. (2021)
*Posthodiplostomum pacificus*	*Larus californicus*	USA	A	MZ710967		MZ707194		Achatz *et al*. (2021)
*Posthodiplostomum anterovarium*	*Lepomis cyanellus*	USA	M	MZ710940		MZ707166, MZ707167		Achatz *et al*. (2021)
*Posthodiplostomum anterovarium*	*Pelecanus erythrorhynchos*	USA	A	MZ710943, MZ710944		MZ707168		Achatz *et al*. (2021)
*Posthodiplostomum ptychocheilus*	*Mergus merganser*	USA	A	MZ710974		MZ707201		Achatz *et al*. (2021)
*Posthodiplostomum minimum*	*Ardea herodias*	USA	A	MZ710961		MZ707190		Achatz *et al*. (2021)
*Posthodiplostomum minimum*	*Nycticorax nycticorax*	USA	A	MZ710962		MZ707191		Achatz *et al*. (2021)
*Posthodiplostomum centrarchi*	*Megaceryle alcyon*	USA	A	MZ710954				Achatz *et al*. (2021)
*Posthodiplostomum centrarchi*	*Ardea herodias*	USA	A	MZ710949				Achatz *et al*. (2021)
*Posthodiplostomum centrarchi*	*Anhinga anhinga*	USA	A	MZ710946, MZ710948				Achatz *et al*. (2021)
*Posthodiplostomum centrarchi*	*Ambloplites rupestris*	USA	M	MZ710945				Achatz *et al*. (2021)
*Posthodiplostomum podicipitis*	*Catostomus commersonii*	USA	M	MZ710968				Achatz *et al*. (2021)
*Posthodiplostomum podicipitis*	*Lophodytes cucullatus*	USA	A	MZ710969, MZ710970		MZ707196, MZ707197		Achatz *et al*. (2021)
*Posthodiplostomum recurvirostrae*	*Recurvirostra americana*	USA	A	MZ710975		MZ707202		Achatz *et al*. (2021)
*Posthodiplostomum* sp. 11	*Chrosomus eos*	USA	M			MZ707203, MZ707204		Achatz *et al*. (2021)
*Posthodiplostomum* sp. 17	*Lophodytes cucullatus*	USA	A	MZ710978		MZ707205		Achatz *et al*. (2021)
*Posthodiplostomum* sp. 18	*Pelecanus erythrorhynchos*	USA	A	MZ710981		MZ707208		Achatz *et al*. (2021)
*Posthodiplostomum* sp. 19	*Physa* sp.	USA	C	MZ710982		MZ707209		Achatz *et al*. (2021)
*Posthodiplostomum* sp. 20	*Physella gyrina*	USA	C			MZ707210, MZ707211		Achatz *et al*. (2021)
*Posthodiplostomum* sp. 21	*Tigrisoma lineatum*	Brazil	A	MZ710989		MZ707212		Achatz *et al*. (2021)
*Posthodiplostomum* sp. 21	*Jabiru mycteria*	Brazil	A			MZ707213	MZ707213	Achatz *et al*. (2021)
*Posthodiplostomum* sp. 22	*Ardea cocoi*	Brazil	A	MZ710992		MZ707215	MZ707214, MZ707215	Achatz *et al*. (2021)
*Posthodiplostomum* sp. 22	*Tigrisoma lineatum*	Brazil	A			MZ707216		Achatz *et al*. (2021)
*Posthodiplostomum* sp. 23	*Ardea herodias*	USA	A	MZ710995		MZ707217	MZ707217	Achatz *et al*. (2021)
*Posthodiplostomum nanum*	*Ardea alba*	USA	A	MZ710963		MZ707192		Achatz *et al*. (2021)
*Posthodiplostomum nanum*	*Gundlachia ticaga*	Brazil	C		MH358392	MH355582		López-Hernández *et al*. (2018)
*Posthodiplostomum nanum*	*Poecilia reticulata*	Brazil	M		MH358393			López-Hernández *et al*. (2018)
*Posthodiplostomum brevicaudatum*	*Perca fluviatilis*	Czech Republic	M	KX931426		KX931418		Stoyanov *et al*. (2017)
*Posthodiplostomum brevicaudatum*	*Gasterosteus aculeatus*	Bulgaria	M		KX931439	KX931420		Stoyanov *et al*. (2017)
*Posthodiplostomum scardinii*	*Scardinius erythrophthalmus*	USA	M	KX931427				Stoyanov *et al*. (2017)
*Posthodiplostomum scardinii*	*Scardinius erythrophthalmus*	Czech Republic	M			KX931425		Stoyanov *et al*. (2017)
*Posthodiplostomum scardinii*	*Ampullaceana balthica*	Denmark	C		MW001051			Duan *et al*. (2021)
*Posthodiplostomum centrarchi*	*Lepomis gibbosus*	Bulgaria, Slovakia	M		KX931441, KX931442/ MF171004			Stoyanov *et al*. (2017)/Kvach *et al*. (2017)
*Posthodiplostomum centrarchi*	*Lepomis gibbosus*	Hungary	M		MN080282- MN080284			Unpublished
*Posthodiplostomum centrarchi*	*Lepomis gibbosus*	Canada	M		HM064953- HM064955			Locke *et al*. (2010)
*Posthodiplostomum* sp.	*Channa punctatus*	India	M	KF738450				Unpublished
*Posthodiplostomum* sp.	*Channa argus*	Japan	M	AB693170				Nguyen *et al*. (2012)
*Posthodiplostomum* sp. 1	*Trichopodus trichopterus*	Vietnam	M	MT394051				Sokolov and Gordeev (2020)
*Posthodiplostomum* sp. 2	*Channa striata*	Vietnam	M	MT394045				Sokolov and Gordeev (2020)
*Posthodiplostomum* sp. 1	*Percina caprodes*	Canada	M		HM064936, HM064937	HM064735		Locke *et al*. (2010)
*Posthodiplostomum* sp. 2	*Notemigonus crysoleucas*	Canada	M		HM064939			Locke *et al*. (2010)
*Posthodiplostomum* sp. 3	*Pimephales promelas*	Canada	M		HM064941, HM064942	HM064780		Locke *et al*. (2010)
*Posthodiplostomum* sp. 4	*Pimephales promelas*	Canada	M		HM064944, HM064945			Locke *et al*. (2010)
*Posthodiplostomum* sp. 5	*Lepomis gibbosus*	Canada	M		HM064958	HM064857		Locke *et al*. (2010)
*Posthodiplostomum* sp. 7	*Perca flavescens*	Canada	M			HM064865		Locke *et al*. (2010)
*Posthodiplostomum* sp. 8	*Pimephales promelas*	Canada	M		HM064946			Locke *et al*. (2010)
*Posthodiplostomum* sp. 8	*Micropterus salmoides*	USA	M		MG857110-MG857112	MG873439, MG873406		Boone *et al*. (2018)
*Posthodiplostomum* sp. 8	*Micropterus dolomieu*	Puerto Rico	M			OP071220, OP071222, OP071223		Pernett *et al*. (2022)
*Posthodiplostomum* sp. 9	*Tilapia sparrmanii*	South Africa	M	MK604823	MK604881			Hoogendoorn *et al*. (2019)
*Posthodiplostomum* sp. 23	*Poecilia reticulata*	Puerto Rico	M			OP071188		Pernett *et al*. (2022)
*Posthodiplostomum* sp. 23	*Gambusia affinis*	Puerto Rico	M			OP071191		Pernett *et al*. (2022)
*Posthodiplostomum* sp. 24	*Poecilia reticulata*	Puerto Rico	M			OP071201, OP071203		Pernett *et al*. (2022)
*Posthodiplostomum* sp. 25	*Dajaus monticola*	Puerto Rico	M			OP071207, OP071208		Pernett *et al*. (2022)
*Crassiphiala* sp. Lineage 5	*Megaceryle torquata*	Brazil	A			MN193959		Unpublished
*Uvulifer spinatus*	*Poecilia mexicana*	México	M	MF568582				López-Jiménez *et al*. (2018)
*Uvulifer spinatus*	*Poeciliopsis* sp.	México	M		MF568657			López-Jiménez *et al*. (2018)
*Uvulifer* sp. 1	*Megaceryle alcyon*	México	A				MF568659	López-Jiménez *et al*. (2018)
*Uvulifer* sp. 2	*Hypsophrys* sp.	México	M				MF568665	López-Jiménez *et al*. (2018)
*Uvulifer* sp. 3	*Cribroheros longimanus*	México	M				MF568672	López-Jiménez *et al*. (2018)
*Uvulifer weberi*	*Chloroceryle americana*	Brazil	A			MK871335		Achatz *et al*. (2019)
*Uvulifer prosocotyle*	*Megaceryle torquata*	Brazil	A			MK871334	MK871334	Achatz *et al*. (2019)
*Posthodiplostomoides kinsellae*	*Halcyon malimbica*	Uganda	A	MZ710939				Achatz *et al*. (2019)
*Neodiplostomum americanum*	*Bubo virginianus*	USA	A	KY851307				Woodyard *et al*. (2017)
*Neodiplostomum americanum*	*Megascops asio*	USA	A		KY851309			Woodyard *et al*. (2017)
*Tylodelphys aztecae*	*Skiffia lermae*	México	M		KT175380			García-Varela *et al*. (2016)
*Tylodelphys aztecae*	*Podilymbus podiceps*	México	A	MF398337				Hernández-Mena *et al*. (2017)
*Parastrigea plataleae*	*Platalea ajaja*	México	A	MF398346	JX977836			Hernández-Mena *et al*. (2017)
*Apharyngostrigea* sp.	*Cnesterodon decemmaculatus*	Argentina	M			MH777790		López-Hernández *et al*. (2019)
*Australapatemon niewiadomski*	*Anas platyrhynchos*	New Zealand	A	KT334165	KT334174			Blasco-Costa *et al*. (2016)
*Diplostomum huronense*	*Catostomus commersoni*	Canada	M		AY123043			Galazzo *et al*. (2002)
*Diplostomum indistinctum*	*Moxostoma anisurum*	Canada	M		AY123044			Galazzo *et al*. (2002)
*Bolbophorus* sp. 3	*Tilapia sparrmanii*	South Africa	M			MK605689		Hoogendoorn *et al*. (2019)

Sequences in bold were obtained in this study. A (adult), M (metacercariae), C (cercaria).

a Previously included in *Posthodiplostomum* sp. lineage V (sensu Pérez Ponce de León *et al*., 2022).

Specimens preserved in 100% ethanol were stained with Mayer's paracarmine (Merck, Darmstadt, Germany), dehydrated in a graded ethanol series, cleared with methyl salicylate and mounted on permanent slides with Canada balsam. Specimens were photographed and measured using a Leica DM 1000 LED compound microscope (Leica Microsystems CMS GmbH, Wetzlar, Germany); measurements are reported in micrometres (*μ*m). Internal morphological features were illustrated using a drawing tube attached to a Leica MC120HD microscope. Drawings were made using Adobe Illustrator 27.9 (Adobe, Inc., CA, USA). Voucher specimens were deposited in the Colección Nacional de Helmintos (CNHE), Instituto de Biología, Universidad Nacional Autónoma de Mexico (UNAM), Mexico City.

Additionally, some specimens preserved in 4% formalin were dehydrated in a graded ethanol series, critical point dried, sputter-coated with gold and examined with a Hitachi Stereoscan Model S-2469N scanning electron microscope at 15 kV at LaNABIO, Instituto de Biología, UNAM.

### Molecular study

Prior to extraction of the genomic DNA, specimens preserved in 100% ethanol were mounted on a microscope slide, and images were taken as references with a bright field Leica DM 1000 LED microscope (Leica, Wetzlar, Germany). Each image was linked with its genomic DNA, (*photogenophore sensu* Andrade-Gómez and García-Varela, 2021). Specimens were removed from the microscope slide and genomic DNA was isolated, following the protocol described by González-García *et al*. (2020). The 28S, ITS1–5.8S–ITS2 and *cox1* genes were amplified by polymerase chain reactions (PCR). The 28S amplifications used forward primer 391, 5′-AGCGGAGGAAAAGAAACTAA-3′ (Nadler *et al*., 2000), and reverse primer 536, 5′-CAGCTATCCTGAGGGAAAC-3′ (Garcia-Varela and Nadler, 2005). The ITS amplifications used forward primer BD1 5′-GTCGTAACAAGGTTTCCGTA-3′ (Bowles and McManus, 1993) and the reverse primer BD2 5′-ATCTAGACCGGACTAGGCTGTG-3′ (Bowles *et al*., 1995). The *cox1* gene was amplified in 2 overlapping fragments. The first region amplifications used forward primer PosthoCoiF, 5′-ATGATWTTTTTTTTYYTRATGCC-3′ and reverse primer PosthoSec1 5′-AAADGAAGAACCRAAWTTHCGATC-3′. The second region amplifications used forward JB3, 5′-TTTTTTGGGCATCCTGAGGTTTAT-3′ and the reverse primer JB4, 5′-TAAAGAACATAATGAAATTG-3′ (Bowles and McManus, 1993).

PCR reactions (25 *μ*L) consisted of 1 *μ*L of each primer (10 *μ*m), 2.5 *μ*L of 10× buffer, 1.5 *μ*L of 2 mm MgCl_2_, 0.5 *μ*L of dNTPs (10 mm), 16.375 *μ*L of water, 2 *μ*L of genomic DNA and 0.125 *μ*L of Taq DNA polymerase (Platinum Taq, Invitrogen Corporation, São Paulo, Brazil). PCR cycling conditions amplifications included initial denaturation at 94°C for 3 min, followed by 35 cycles of 1 min at 94°C, 1 min at 48°C for fist region of *cox1*, 45°C for second region of *cox1* and 50°C for ITS1–5.8S rDNA–ITS2 and 28S, and 1 min at 72°C; followed by a final 10 min at 72°C. Sequencing reactions were performed using ABI Big Dye (Applied Biosystems, Boston, MA, USA) terminator sequencing chemistry and reaction products were separated and detected using an ABI 3730 capillary DNA sequencer. Contigs were assembled, base-calling differences resolved using Codoncode Aligner version 9.0.1 (Codoncode Corporation, Dedham, MA, USA) and submitted to the GenBank (Table 1).

### Alignments and phylogenetic analyses

Newly generated sequences of 28S, ITS1–5.8S–ITS2 and *cox1* were aligned with other diplostomid sequences available in GenBank (Table 1). Sequences of each molecular marker were aligned using SeaView version 4 (Gouy *et al*., 2010) and adjusted with Mesquite program (Maddison and Maddison, 2011). The nucleotide substitution model was selected using jModelTest v2.1.7 (Darriba *et al*., 2012) applying the Akaike information criterion. The best nucleotide substitution model for 28S and ITS dataset was TVM + I + G and for both regions of *cox1* was GTR + G + I.

Phylogenetic analyses were reconstructed through Bayesian inference (BI) and maximum likelihood (ML) using the online interface Cyberinfrastructure for Phylogenetic Research (CIPRES) Science Gateway v3.3 (Miller *et al*., 2010). BI analysis was inferred with MrBayes v.3.2.7 (Ronquist *et al*., 2012), with 2 simultaneous runs of the Markov Chain Monte Carlo (MCMC) for 10 million generations, sampled every 1000 generations, using a heating parameter value of 0.2 and a burn-in of 25%. ML analysis was carried out with RAxML v.7.0.4 (Silvestro and Michalak, 2011), and 1000 bootstrap replicates were run to assess nodal support. Phylogenetic trees were drawn and edited in FigTree v.1.3.1 (Rambaut, 2012). Genetic divergence among taxa was estimated using uncorrected ‘*p*’ distances with MEGA6 (Tamura *et al*., 2013).

## Results

### Phylogenetic analyses

#### Nuclear genes

**Figure 2.**
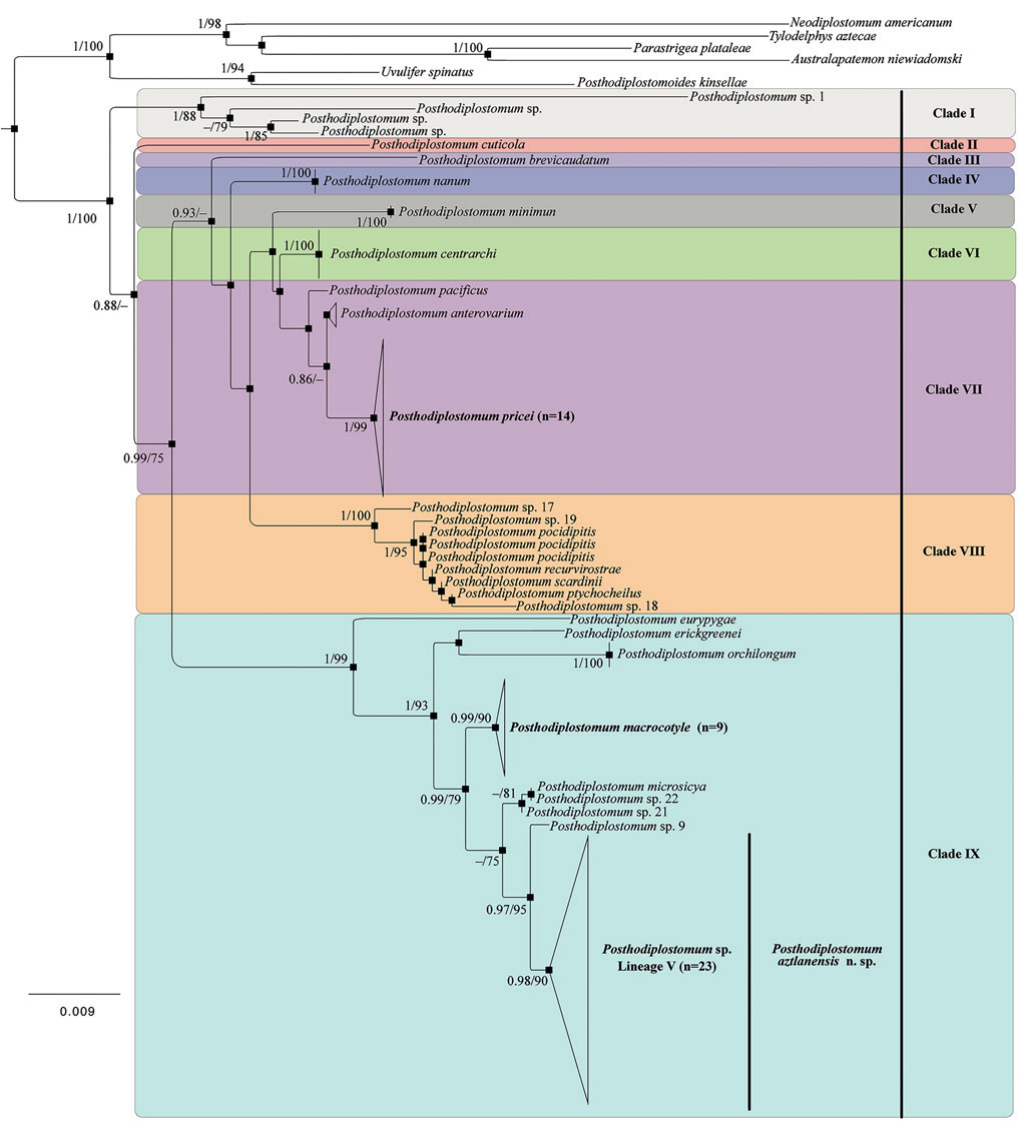
Phylogenetic trees inferred with maximum likelihood (ML) and consensus Bayesian inference (BI) of 28S from nuclear ribosomal DNA. Numbers near internal nodes show maximum likelihood bootstrap percentage values and Bayesian posterior probabilities. Sequences generated in this study in bold. Clades highlighted in pink and blue are equivalent in the phylogenetic trees inferred with internal transcribed spacers from nuclear ribosomal DNA (Fig. 4).

**Figure 3.**
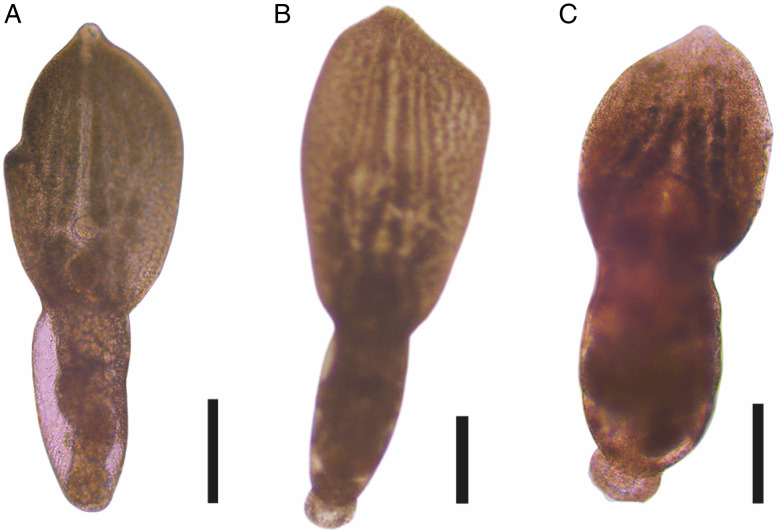
Photogenophores of *Posthodiplostomum macrocotyle*. Specimens collected in Emiliano Zapata, Tabasco, Mexico from *Tigrisoma mexicanum* (A); *Leucophaeus atricilla* (B); *Ardea herodias* (C). Scale bars: 200 *μ*m.

The 42 newly generated (28S) sequences were analysed together with 42 sequences of *Posthodiplostomum* spp. plus sequences of 6 species of diplostomids used as outgroups (Table 1). The alignment comprised 90 sequences with 1098 characters after trimming to the shortest sequence. The phylogenetic analyses identified *Posthodiplostomum* as a monophyletic assemblage with strong bootstrap support (100%) and a strong Bayesian posterior probability (1.0) (Fig. 2). The phylogenetic trees revealed 9 main clades (Fig. 2). The first clade contained sequences of *Posthodiplostomum* sp. metacercariae from the Indomalayan and Palaearctic regions. Clades II–VI formed a single lineage representing the following species: *P. cuticola* von Nordmann, 1832; *P. brevicaudatum* von Nordmann, 1832; *P. nanum* Dubois, 1937; *P. minimum*; and *P. centrarchi* Hoffman, 1958 (Fig. 2). Clade VII included sequences of *P. pacificus* Achatz *et al*., 2021, and *P. anterovarium* Dronen, 1985, and 12 new sequences of adult specimens from *Rynchops niger* L, from Campeche, Mexico (locality 3 in Fig. 1), which nested with 2 sequences (MZ710972–MZ710973), identified as *P. pricei* (Krull, 1934), from *Larus delawarensis* Ord., from North Dakota, USA. Clade VIII included sequences of unidentified species of *Posthodiplostomum* sp.; *P. podicipitis* Yamaguti, 1939; *P. recurvirostrae* Achatz *et al*., 2021; *P. scardinii* Shulman, 1952; and *P. ptychocheilus* Faust, 1917. Finally, clade IX consisted of 6 subclades. One of them included 2 sequences previously identified as *P. macrocotyle* Dubois, 1937 (MZ710958–MZ710959) from Brazil nested with 7 new sequences from adult specimens (Fig. 3) (*Tigrisoma mexicanum* Swainson, *Ardea herodias* L. and *Leucophaeus atricilla* L.) from Tabasco, Mexico (locality 4 in Fig. 1). Another subclade included 22 newly sequenced individuals from *A. herodias*, *Butorides virescens* L, *N. nycticorax* L and *T. mexicanum* sampled in 3 localities of Mexico (localities 1, 2 and 4 in Fig. 1), plus 1 sequence from a poecilid fish from Las Brisas del Chamalecon, Honduras, identified as *Posthodiplostomum* sp. lineage V (*sensu* Pérez-Ponce de León *et al*., 2022). This clade represents a new species described herein as *Posthodiplostomum aztlanensis* n. sp. (Fig. 2).

**Figure 4.**
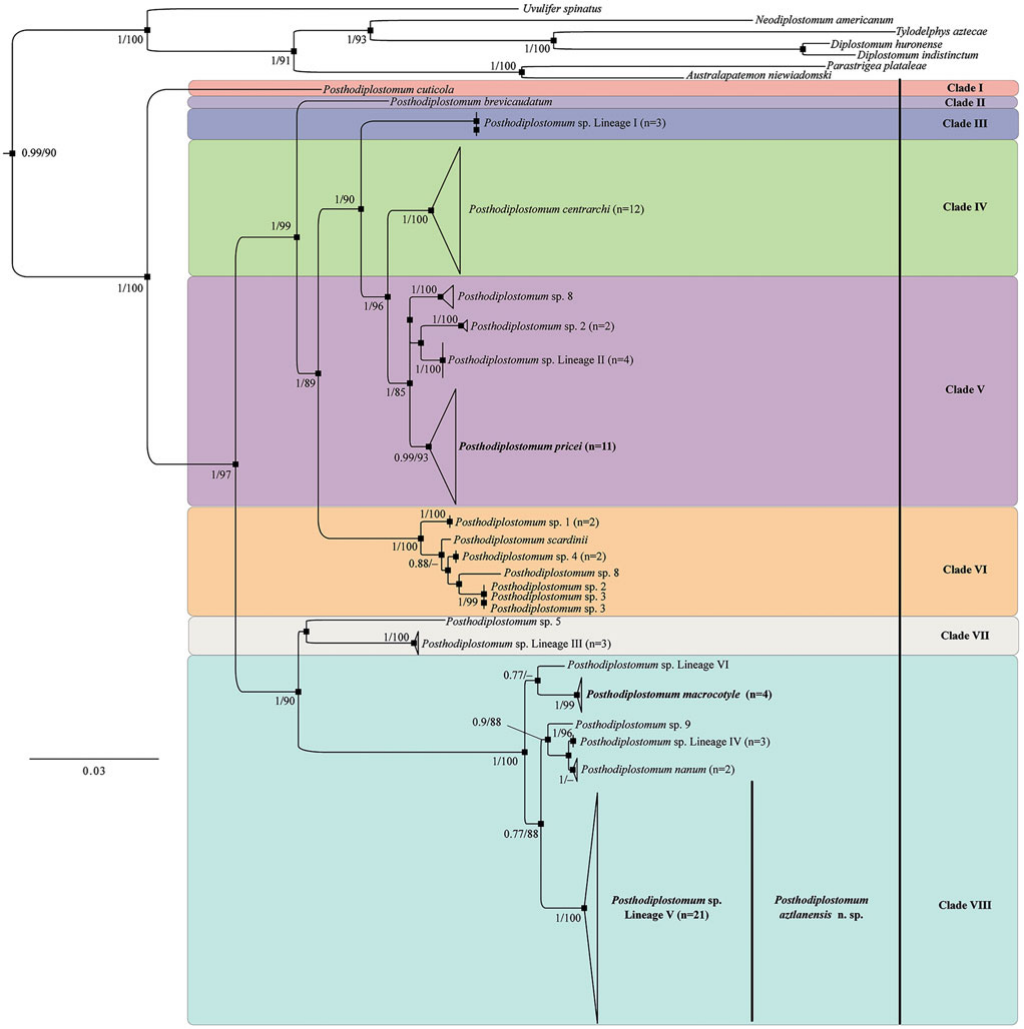
Phylogenetic trees inferred with maximum likelihood (ML) and consensus Bayesian inference (BI) of ITS1–5.8S–ITS2 from nuclear ribosomal DNA. Numbers near internal nodes show maximum likelihood bootstrap percentage values and Bayesian posterior probabilities. Sequences generated in this study in bold. Clades highlighted in pink and blue colours are equivalent in the phylogenetic trees inferred with the large subunit from nuclear ribosomal DNA (Fig. 2).

The 22 newly generated ITS sequences were analysed together with 60 sequences of *Posthodiplostomum* spp., plus 7 sequences from other diplostomids downloaded from the GenBank dataset that were used as outgroups (Table 1). The ITS1–5.8S–ITS2 alignment consisted of 89 sequences with 1100 characters after trimming to the shortest sequence. The phylogenetic analyses inferred with the ITS dataset also revealed the monophyly of *Posthodiplostomum* (Fig. 4). In particular, clade V included sequences of *Posthodiplostomum* sp. 8, 2 (*sensu* Locke *et al*., 2010), and *Posthodiplostomum* sp. lineage II (*sensu* Pérez-Ponce de León *et al*., 2022), plus 9 new sequences of *Posthodiplostomum* spp. from Campeche, Mexico (locality 3 in Fig. 1), nested with 2 sequences previously identified as *P. pricei* (HM064959–HM064960) from the white perch (*Morone americana* Gmelin) from Canada (Fig. 4), showing conspecificity. Clade VIII was formed by *Posthodiplostomum* sp. 9 (*sensu* Hoogendoorn *et al*., 2019), *Posthodiplostomum* sp. lineage IV and VI (*sensu* Pérez-Ponce de León *et al*., 2022), and *P. nanum* plus 4 new sequences identified as *P. macrocotyle* from 3 host species (Fig. 4) (*T. mexicanum*, *A. herodias* and *L. atricilla*) from Tabasco, Mexico (locality 4 in Fig. 1). The sister subclade of the latter consisted of 19 sequences representing the new species from 3 localities across Mexico (including 3 sequences of metacercariae identified as *Posthodiplostomum* sp. lineage V (*sensu* Pérez-Ponce de León *et al*., 2022) from poecilids, goodeids and eleotrids from Honduras, Mexico and Costa Rica (Fig. 4).

#### Mitochondrial gene

**Figure 5.**
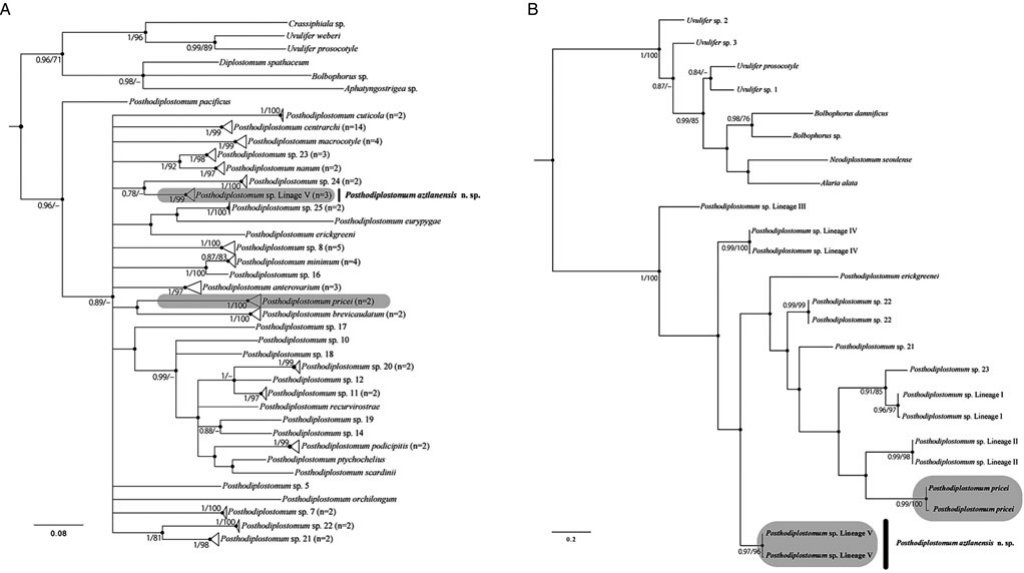
Phylogenetic trees inferred with maximum likelihood (ML) and consensus Bayesian inference (BI) mitochondrial cytochrome c oxidase subunit 1 (*cox1*) genes. The first region of the *cox1* (A). The second region of the c*ox1* (B). Numbers near internal nodes show maximum likelihood bootstrap percentage values and Bayesian posterior probabilities.

For the *cox1* gene, 2 datasets were used. The first included the *cox1* barcoding region. This dataset included 5 new sequences, 80 sequences of *Posthodiplostomum* spp., plus 6 sequences of diplostomids as an outgroup. The alignment was 553 bp long. With ML and BI, phylogenetic analyses identified *Posthodiplostomum* as monophyletic, although with moderate posterior probability and low bootstrap support values. Furthermore, *P. pacificus* was identified as the sister taxon of an unresolved clade that included all the remaining species/lineages of *Posthodiplostomum* (Fig. 5A). Three sequences of the new species nested with sequences of lineage V (*sensu* Pérez-Ponce de León *et al*., 2022). The other 2 sequences nested with *P. pricei*. The second alignment included approximately 380 bp, which corresponds to the 3′ region of the *cox1* gene. The topology of the tree is better resolved, although it contains a small number of sequenced individuals. This dataset contained 4 new sequences, 12 sequences of *Posthodiplostomum* spp. plus 8 diplostomids used as an outgroup. The tree also revealed the monophyly of *Posthodiplostomum* as well as the monophyly of the 4 new sequences; 2 belonged to *P. pricei*, and the other 2 corresponded to the new species (Fig. 5B).

### Genetic divergence

The 28S intraspecific genetic divergence among 14 isolates of *P. pricei* was very low, ranging from 0 to 0.09%, whereas that among 9 isolates of *P. macrocotyle* ranged from 0 to 0.18%, and that among 23 isolates of *P. aztlanensis* n. sp. ranged from 0 to 0.45% (Supplementary Table 1). The interspecific divergence among *Posthodiplostomum* spp. varied between 0 and 7.86%; the greatest divergence was found between 1 isolate of *P. macrocotyle* from *L. atricilla* in Tabasco, Mexico, and *Posthodiplostomum* sp. 1 from *Trichopodus trichopterus* Pallas, in Vietnam (MT394051). The interspecific divergence between the new species and all congeners varied from 0.63 to 5.23%.

The intraspecific genetic divergence of the ITS region among the 11 *P. pricei* isolates was also low, ranging from 0 to 0.78%; the greatest difference was found between 1 isolate (HM064959) from *M. americana* in Canada and 1 isolate from *R. niger* in Campeche (locality 3 in Fig. 1), whereas the divergence among the 4 *P. macrocotyle* isolates ranged from 0 to 0.11% and among the 21 *P. aztlanensis* n. sp. isolates ranged from 0 to 0.38%. The interspecific genetic divergence of the ITS region between the new species and all other species ranged from 1.18 to 11.7% (Supplementary Table 2).

Finally, the *cox1* intraspecific genetic divergence among isolates of *P. pricei* ranged from 0 to 2.6%, and among isolates of *P. aztlanensis* n. sp., the divergence varied from 0.47 to 0.94% and 0.53% from the first and second regions of *cox1*, respectively. For the interspecific genetic divergence of *cox1*, 2 values were obtained, 1 for each database (Supplementary Tables 3 and 4). The largest interspecific genetic divergence for the first region of *cox1* of *Posthodiplostomum* spp. ranged from 19 to 22.3% between *P. pricei* and *P. cuticola*, whereas for the second region of *cox1*, it ranged from 18.6 to 19.3% between *P. aztlanensis* and *Posthodiplostomum* lineage II.

### Morphological description

#### Family Diplostomidae Poirier, 1886 Genus *Posthodiplostomum* Dubois, 1936

*Posthodiplostomum aztlanensis* n. sp.

*Type host*: *Butorides virescens* (Little Green Heron) (Pelecaniformes: Ardeidae).

*Other hosts*: *Ardea herodias* (Great Blue Heron) (Ardeidae); *Nycticorax nycticorax* (black-crowned Night Heron) (Ardeidae); *T. mexicanum* (bare-throated Tiger-Heron) (Ardeidae).

*Type locality*: Marquelia, Guerrero, Mexico (16°35′41.5″N, 98°50′38″W).

*Other localities*: Emiliano Zapata, Tabasco, Mexico (17°46′29.1″N, 91°44′24.9″W); Tlacotalpan, Veracruz, Mexico (18°36′0″N, 95°39′0″W).

*Site in host*: Intestine

*Type material*: Holotype CNHE: 12990; paratypes CNHE: 12991–12993

*GenBank accession number*: 28S: PP718597–PP718619; ITS: PP718639–718656; *cox1*: PP724755–PP724757.

*ZooBank registration*: To comply with the regulations set out in article 8.5 of the amended 2012 version of the International Code of Zoological Nomenclature (ICZN, 2012), details of the new species have been submitted to ZooBank. The Life Science Identifier (LSID) for *P. aztlanensis* n. sp. is urn:lsid:zoobank.org:act:E12C7AD7-BB6D-411B-A3BC-43D38ADB71C0

*Etymology*: The epithet is dedicated to the city of ‘Aztlan’, where the Aztec culture originated, which in Nahuatl means place of herons.

#### Description (Fig. 6; Table 2)

**Figure 6.**
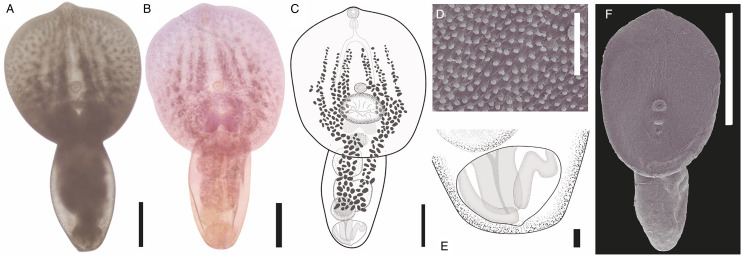
*Posthodiplostomum aztlanensis* n. sp. collected from *Butorides virescens* in Marquelia, Guerrero, Mexico. Ventral view of the *photogenophore* (A); photograph of the holotype (B); ventral view of the holotype (C); scanning electron micrograph, tegument spines (D); posterior end of the holotype and genital cone (E); whole worm (F). Scale bars: (A, B, C) 200 *μ*m; (D) 10 *μ*m; (E) 20 *μ*m; (F) 400 *μ*m.

Description (based on 33 adult specimens); measurements of holotype (Fig. 6B) given in text; measurements of the entire series given in Table 2. Body 1096 long, consisting of distinct prosoma and opisthosoma (Fig. 6C); prosoma oval, 676 long, widest at mid-length, 614 wide. Opisthosoma cylindrical, 502 long, much narrower than prosoma, 276 wide. Prosoma: opisthosoma length ratio 1:1.3. Tegument completely armed with pectinate spines (Fig. 6D). Oral sucker terminal, 48 (length) × 54 (width). Ventral sucker equal size to oral sucker, 46 × 54, post equatorial of prosoma. Oral: ventral sucker ratio 1:1.05 × 1:0.98. Holdfast organ immediately posterior to ventral sucker, oval transversely elongated with ventral muscular portion, 157 × 201. Proteolytic gland dorsal to posterior part of holdfast organ, bilobed. Prepharynx not observed. Pharynx oval, 46 × 36. Oesophagus larger than pharynx, 55 long. Caecal bifurcation in the most anterior quarter of prosoma length. Caeca slender, end not observed due to vitellarium.

**Table 2.** Comparative measurements of adult specimens of *Posthodiplostomum aztlanensis* n. sp. and *Posthodiplostomum pricei*

	*Posthodiplostomum aztlanensis* n. sp.	*Posthodiplostomum pricei*	*Posthodiplostomum pricei*
Features	*n* = 30	*n* = 12	
Host	*Butorides virescens*, *Ardea herodias*, *Nycticorax nycticorax*, *Tigrisoma mexicanum*	*Rynchops niger*	*Larus novaehollandiae*,^a^ *Larus argentus*, *Larus delawarensis*, *Larus philadelphia*
Locality	Mexico	Mexico	United States
Source	Present study	Present study	Krull (1934); Dubois (1970)
Body length	779–1392 (978)	1246–1679 (1402)	2342^b^–2500
Prosoma length	449–938 (616)	835–1154 (927)	1020–1600 (1400)
Prosoma width	279–614 (463)	213–367 (264)	360–665 (632)
Opisthosoma length	303–554 (412)	436–622 (509)	550–960 (748)
Opisthosoma width	174.5–359 (254)	238–320 (277)	300–520 (472)
Prosoma: opisthosoma length ratio	1–2.18 (1.53)	1.5–2.3 (1.8)	–
Prosoma (% of body length)	51–67 (62)	60–71 (66)	–
Oral sucker length	39–56 (47)	27–40 (35)	37–60 (47)
Oral sucker width	33–61 (45)	23–30 (26)	30–44 (38)
Ventral sucker length	30.6–79.6 (44)	48–70 (62)	55–92 (72)
Ventral sucker width	37–99 (50)	43–65 (56)	73–112 (91)
Oral sucker: ventral sucker width ratio	0.61–1.24 (0.94)	0.38–0.69 (0.48)	–
Oral sucker: ventral sucker length ratio	0–94–1.51 (1.11)	0.43–0.83 (0.57)	–
Holdfast organ length	77–182 (109)	121–163 (137)	150–270
Holdfast organ width	86–201 (131)	70–118 (89)	120–230
Holdfast organ position (% of prosoma length)	12–23.3 (17.7)	11.8–18.5 (14.9)	16^b^
Pharynx length	29–49 (42)	27–40 (33)	33–53 (43)
Pharynx width	26–43 (32)	17–25 (21)	24–41 (33)
Oral sucker: pharynx length ratio	0.88–1.48 (1.13)	0.79–1.23 (1.05)	–
Oesophagus length	34–70 (51.5)	23–84 (42)	–
Anterior testis length	65–149 (103)	105–171 (123)	205–318 (280)
Anterior testis width	89.5–141 (111)	150–208 (182)	250–430 (387)
Posterior testis length	63.5–194 (118)	92–238 (139)	180–360 (330)
Posterior testis width	105–246 (167)	161–244 (206)	280–470 (435)
Ovary length	42–89 (61)	61.5–81.5 (70)	80–140 (120)
Ovary width	48–101 (77)	50–75 (64)	86–140 (104)
Egg length	60–86	58–98	86–92
Egg width	42–67	38–59	66–72
Anterior vitellarium free zone	134–337 (203)	443–684 (514)	800^b^
Anterior vitellarium free zone (% of prosoma length)	23–45.5 (32.9)	49.3–61.8 (55.2)	48^b^
Posterior vitellarium free zone	78–165 (131)	119–193 (145)	216^b^
Posterior vitellarium free zone (% of opisthosoma length)	20.5–40 (33)	22.2–34.2 (28.5)	24.9^b^

Measurements in micrometres. ^a^Host experimental. ^b^Estimated from the published drawing (Krull, 1934).

Testes 2, tandem; anterior testis positioned posterior to prosoma end, subspherical 149 × 141, posterior testis somewhat bilobed, 163 × 167. Seminal vesicle post-testicular, ventral to posterior testis, compact, continues to short ejaculatory duct. Ejaculatory duct joins metraterm to form hermaphroditic duct. Hermaphroditic duct opening at genital cone into genital atrium; genital cone surrounded by prepuce within genital atrium. Genital pore terminal (Fig. 6E).

Ovary pretesticular, posterior part of ovary ventral to anterior testis, transversely oval, positioned near prosoma–opisthosoma junction and posterior to proteolytic gland, 70 × 92. Oötype and Mehlis' gland not observed. Laurer's canal not observed. Vitellarium located from near caecal bifurcation in prosoma, extending to opisthosoma to the posterior margin of testis. Eggs not observed. Excretory vesicle not observed. Excretory pore subterminal.

### Remarks

*Posthodiplostomum aztlanensis* n. sp. belongs to genus *Posthodiplostomum* based on the results of our molecular analyses as well as the presence of a genital prepuce and lack of pseudosuckers. The new species can be distinguished from all other *Posthodiplostomum* spp., except for *Posthodiplostomum biellipticum* Dubois, 1958 and *Posthodiplostomum grayi* (Verma, 1936), by its prosoma shape (oval), whereas variable form in all other *Posthodiplostomum* spp. (concave, linguiform or lanceolate). The new species and *P. biellipticum* can be further distinguished based on the prosoma: opisthosoma length ratio (opisthosoma being longer in *P. biellipticum* than *P. aztlanensis*). In addition, both species *P. biellipticum* and *P. grayi* can be distinguished based on length of body (1450 in *P. biellipticum* and *P. grayi*, *vs* 779–1392 in *P. aztlanensis*). The biogeographical distribution can be used as another character to distinguish the species. For example, *P. biellipticum* has been recorded in Ghana (Africa), *P. grayi* in India, China, Philippines (Asia), whereas *P. aztlanensis* was recorded in the Neotropical region of Mexico (Americas).

### Morphological identification

#### *Posthodiplostomum pricei* (Krull, 1934)

*Host*: *Rynchops niger* (Black Skimmer) (Charadriiformes: Laridae).

*Locality:* Nuevo Campechito, Campeche, Mexico (18°38′55.849″N, 92°28′2.578″W).

*Site in host*: Intestine

*Voucher*: CNHE: 12994

*GenBank accession number*: 28S:PP718620–PP718631; ITS: PP718657–PP718665; *cox1*: PP724758–PP724759.

**Figure 7.**
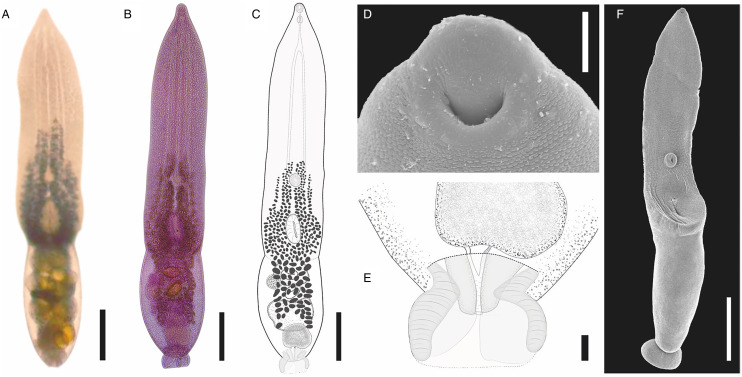
*Posthodiplostomum pricei* collected from *Rynchops niger* in Nuevo campechito, Campeche, Mexico, ventral view of the *photogenophore* (A); photograph of the vouchers (B); ventral view (C); scanning electron micrograph, oral sucker and tegument spines (D); posterior end of the voucher and genital cone (E); whole worm (F). Scale bars: (A, B, C, F) 200 *μ*m; (D) 10 *μ*m; (E) 20 *μ*m.

Sixteen adult specimens were collected, measured and compared with described species. Our specimens were morphologically identified as *P. pricei*; overall, specimens are similar to those described of *P. pricei* by Krull (1934) in the original description, and redescribed later by Dubois (1970). In addition, the genetic data generated in this study supported the morphological evidence, confirming that all the specimens belong to *P. price*. Our specimens are similar to those descriptions for the prosoma shape (lanceolate), ovary position (intertesticular), the prosoma:opisthosoma length ratio, prosoma:body length ratio, the holdfast:prosoma length ratio, the oral sucker:pharynx length ratio and the oesophagus length. Our specimens are, however, smaller than those from previous descriptions (Fig. 7; Table 2).

## Discussion

Adults of the genus *Posthodiplostomum* are known to infect the intestines of fish-eating birds, mainly those of the family Ardeidae (Ritossa *et al*., 2013; López-Hernández *et al*., 2018; Perez-Ponce de León *et al*., 2022; Achatz *et al*., 2021). López-Hernández *et al*. (2018) suggested that species of *Posthodiplostomum* have diversified in the Neotropical region. More recently, Pérez-Ponce de León *et al*. (2022) assessed the diversity of the genus through an analysis of the genetic variation of metacercariae in freshwater fishes across Middle America (Mexico, Guatemala, El Salvador, Honduras and Costa Rica). These authors sequenced 2 molecular markers, the internal transcribed spacer (ITS1–5.8S–ITS2) and 1 region of the mitochondrial *cox1* gene. Their molecular analyses yielded 6 genetic lineages that did not correspond to any available sequences of *Posthodiplostomum* in GenBank at the time. Finally, Pernett *et al*. (2022) suggested that the biodiversity of *Posthodiplostomum* in the Neotropical region was sub estimated. In the present study, adult specimens were collected from fish-eating birds at several locations in the Neotropical region of Mexico. Phylogenetic analyses with 28S, ITS and *cox1* revealed that adults were allocated to 3 independent clades. One of these clades corresponded to lineage V (*sensu* Pérez-Ponce de León *et al*., 2022), and we linked the metacercariae recovered from 3 fish families (Goodeidae Jordan, Eleotridae Bonaparte and Poecilidae Bonaparte). This lineage represented a new species, *P. aztlanensis* n. sp., which seems to be, as adults, host-specific to birds of the family Ardeidae. This represents the first species described in the Neotropical region of Mexico. In addition to morphological evidence and the position of the new lineage in the phylogenetic trees, the genetic divergence found between adults and metacercariae provided additional support for the separation of the species. For example, the intraspecific genetic divergence among isolates was very low (0–0.45% for 28S, 0–0.38% for ITS, 0.47–0.94% and 0.53% for the first and second regions of *cox1*). This low divergence level, particularly that of *cox*1, is similar to that reported previously by Achatz *et al*. (2021) (less than 4.1% were considered conspecific). The interspecific divergence between the new species and its congeners varied from 0.63 to 5.23% for 28S, 1.18 to 11.7% for ITS, and 10.3 to 19.4% and 10.9 to 19.3% for the first and second regions of *cox1*, respectively. These range values are larger than those previously reported by Achatz *et al*. (2021), which were 4.1%.

Furthermore, molecular analyses were useful for identifying 2 additional species of *Posthodiplostomum*. One of them was *P. macrocotyle*, which was found in 3 bird species (*T. mexicanum*, *A. herodias* and *L. atricilla*) from Tabasco, Mexico (see Fig. 3); these records represent new locality records and expand the distribution range of the species. Newly generated sequences were placed together in a clade with 2 sequences identified as *P. macrocotyle* from the black-collared hawk (*Busarellus nigricollis* Latham) from Brazil (MZ710958–MZ71095, Fig. 2), with a low genetic divergence value (0–0.18%). *Posthodiplostomum macrocotyle* was originally described by Dubois (1937) from specimens recovered from the black skimmer *R. niger* in Brazil. Therefore, the presence of *P. macrocotyle* expands the geographical distribution of the species further north in the Neotropical region. Moreover, *P. macrocotyle* is considered a generalist species since it has been recorded in at least 5 host species belonging to 3 bird families (Accipitridae Vieillot, Laridae and Ardeidae). However, no matches were found between *P. macrocotyle* and the genetic lineages of metacercariae reported in Pérez-Ponce de León *et al*. (2022).

The second species, supported by phylogenetic analyses, genetic divergence and morphological evidence, corresponded to *P. pricei*. The taxonomic history of this taxon has been controversial. The species was originally described as *Neodiplostomum pricei* by Krull (1934) as a parasite of the silver gull *Chroicocephalus novaehollandiae* Stephens in Washington, USA; the species was later transferred to the genus *Mesoophorodiplostomum* by Dubois (1936) and accepted by Niewiadomska (2002). The first sequences of metacercariae from 3 fish species (*Fundulus diaphanous* Lesueur, *F. heteroclitus* L. and *Lepomis gibossus* L.) from Canada were assigned to *Posthodiplostomum* sp. 6 (Moszczynska *et al*., 2009; Locke *et al*., 2010). Later, a sequence from an adult specimen experimentally obtained from the American herring gull (*Larus argentatus* Pontoppidan) was identified as *P. pricei* (see Blasco-Costa and Locke, 2017). More recently, Achatz *et al*. (2021) obtained sequences (28S and *cox1*) from an adult specimen recovered from the ring-billed gull *L. delawarensis* in North Dakota, USA. Their phylogenetic analyses placed *M. pricei* within the genus *Posthodiplostomum* and transferred *M. pricei* to *Posthodiplostomum* as *P. pricei* (Krull, 1934). Additionally, the sequences of metacercariae, referred to as *Posthodiplostomum* sp. 6, were linked with those sequences of Blasco-Costa and Locke (2017) and transferred to *P. pricei* (see Achatz *et al*., 2021). Our specimens from the black skimmer, *R. niger* L., which were sampled in Campeche, Mexico, match all these sequences and expand southwards the distribution range of the species from the Nearctic region to southeastern Mexico in the Neotropical region. In this case, *P. pricei* shows narrow host specificity towards its definitive host (Laridae).

Therefore, considering the 3 species reported in this study, in addition to at least 5 other genetic lineages (candidate species) of the genus *Posthodiplostomum* occurring in Mexico, we could consider it a hotspot of diversity due to its transitional position between the Nearctic and Neotropical biogeographical regions (Morrone, 2006; Pérez-Ponce de León *et al*., 2007). In addition, the results of the present study suggest that the Neotropical region of Mexico meets the ecological requirements to complete the life cycle of *P. aztlanensis* n. sp., *P. macrocotyle* and *P. pricei*, which is key to their distribution. The same pattern of sympatric distribution has been observed in other species of diplostomids, strigeids and clinostomids. For example, *Tylodelphys aztecae* (García-Varela *et al*., 2016) was found in the Neotropical region of Mexico, whereas *Tylodelphys* sp. 6 (*sensu* Locke *et al*., 2015) was initially recorded in the Nearctic region and was later found in the Neotropical region of Mexico (Sereno-Uribe *et al*., 2018); *Strigea macrobursa* (Drago and Lunaschi, 2011) was described in Argentina, and it has been recorded in Mexico, together with *Strigea magnirostris* (López-Jiménez *et al*., 2023). Similarly, *Clinostomum tataxumui* is restricted to the Neotropical region, whereas *Clinostomum marginatum* has been recorded in both the Nearctic and Neotropical regions (Sereno-Uribe *et al*., 2013).

Finally, our study represents a step forward in our comprehension of parasite biodiversity in biogeographical transitional areas and provides new molecular and morphological data to delineate and describe new species of trematodes infecting fish-eating birds. Nevertheless, a larger bird sampling effort is required to increase the genetic library of the trematodes infecting birds to establish a more precise link with the metacercariae found in a diverse array of fish.

## Supporting information

González-García et al. supplementary material 1 González-García et al. supplementary material

González-García et al. supplementary material 2 González-García et al. supplementary material

González-García et al. supplementary material 3 González-García et al. supplementary material

González-García et al. supplementary material 4 González-García et al. supplementary material

## Data Availability

The genetic distances estimated among the taxa for each molecular marker can be download. The alignments can be obtained from the corresponding author upon request.

## Supplementary material

The supplementary material for this article can be found at https://doi.org/10.1017/S0031182024000970
